# Association of parents' alcohol use and family interaction with the initiation of alcohol use by sixth graders: A preliminary study in Taiwan

**DOI:** 10.1186/1471-2458-9-172

**Published:** 2009-06-04

**Authors:** Chao-Chia Hung, Lee-Lan Yen, Wen-Chi Wu

**Affiliations:** 1 Department of Nursing, College of Health Sciences, Yuanpei University, 306 Yuanpei Street, Hsinchu 300, Taiwan, Republic of China; 2 Institute of Health Policy and Management, College of Public Health, National Taiwan University, 17 Hsu-Chow Road, Taipei 100, Taiwan, Republic of China; 3 Center for Health Policy Research and Development, National Health Research Institutes, 35 Keyan Road, Zhunan, Miaoli 350, Taiwan, Republic of China; 4 Department of Public Health, College of Public Health, National Taiwan University, 17 Hsu-Chow Road, Taipei 100, Taiwan, Republic of China

## Abstract

**Background:**

The family is the main environment where children are socialized and learn individual behavior. Although previous studies have examined predictors of preadolescent first alcohol use, few studies have analyzed factors associated with alcohol use in children in a country with low alcohol consumption. The aim of this study was to investigate the initiation of alcohol use by sixth graders and determine family factors associated with first alcohol use.

**Methods:**

Data used in this study was collected as part of the Child and Adolescent Behaviors in Long-term Evolution (CABLE) project in 2002 (when study participants were in grade 5 and aged 10–11 years) and 2003 (when study participants were in grade 6 and aged 11–12 years). Data from a total of 1,183 participants was analyzed. Main study variables included children's alcohol use: (1) never user (never user in 2002 and 2003), or (2) first-time user (never user in 2002 but ever user in 2003); parents' alcohol use: (1) both parents ever users, (2) mother ever user and father never user, (3) father ever user and mother never user, (4) both parents never users; parental support; and family conflict. Correlates of first alcohol use were identified using logistic regression.

**Results:**

There were 183 students (15.5%) who became first-time users of alcohol in the sixth grade. Having parents who both used alcohol, less parental support, and more family conflict were significant predictors of sixth graders' first alcohol use. Family interaction and parents' drinking were equally important predictors of preteen's first use of alcohol.

**Conclusion:**

Family factors influence children's initiation of alcohol use. It is important to educate parents about the effects of alcohol on children and to emphasize the importance of prevention.

## Background

Underage drinking is a long standing major public health issue. According to the Global Status Report [1], in 23 countries in the European Union about half of school children have already tried alcohol by age 11. Youth who start drinking early are at elevated risk of involvement in later alcohol and hard drug abuse [2,3]. Moreover, a number of studies have found that underage drinkers are at an increased risk of experiencing alcohol related unintentional or intentional injures compared to those who begin drinking at a later age [4-9]. The World Health Organization (WHO) [1,10] has emphasized that the drop in age of onset of alcohol use by children and adolescents is an important issue for all nations and that effective strategies to delay the age of first alcohol use are needed. To date most research into factors associated with underage first use of alcohol has been carried out in countries with high alcohol consumption. Compared to Western countries alcohol consumption in Asian countries such as Taiwan is quite low [11,12]. However, as alcohol consumption among underage youth tends to increase along with rapid economic growth and lifestyle westernization [10], first alcohol use is an important issue in a low drinking society with high economic growth.

Although genetic factors contribute to the initiation of alcohol use [13,14], the initiation of alcohol use in early adolescence is strongly influenced by social and familial environmental factors [15]. The social development model [16] incorporates social learning theory and social control theory in an integrated theoretical approach to identifying etiological and developmental mechanisms affecting behavior. Social learning theory [17] emphasizes the effect of modeling behavior. Young people who perceive more people drinking (e.g. parents or peers) are predicted to repeat this behavior in the future. Social control theory [18] emphasizes the predictive effect on behavior of social bonds to parents, school, peers and the community. Bonding is expected to serve a protective function against deviant behavior. The acceptance of alcohol consumption is a socialization behavior. As children are primarily socialized in the family, it is important to identify the familial factors associated with underage alcohol use.

Most studies have found parents' drinking and family interactions to be associated with adolescents' alcohol use. Greater alcohol use by parents is associated with earlier use of alcohol by adolescents [19-22] and children are more likely to use alcohol if both parents drink alcohol, as opposed to only one parent [23]. However, no consistent specific effects of either father's or mother's drinking alone on children's alcohol use have been found. Zhang et al. [24] found that father's drinking had a direct effect on adolescent alcohol use, but that mother's drinking did not. Most studies concerning the effect of parents' drinking on alcohol use either looked at mother's and father's alcohol use separately or did not provide a definition of parents' drinking. Few studies have investigated the impact of different match in problem alcohol use between parents. Moser & Jacob [25] found that children with alcoholic parents were more likely to have problem behavior, regardless of whether alcoholism was present in the mother only, father only, or both parents. Further research is needed into the role of different combinations of parents' drinking on children's first use of alcohol.

Parenting behavior can also moderate child outcomes. One study found that children whose parents communicated more with them when they were in the sixth grade, and gave them more encouragement and physical affection in the seventh grade, were less likely to start using alcohol monthly when they reached the ninth grade [26]. In contrast, in families with more conflict, children and adolescents are more likely to use alcohol [27,28]. Hawkins et al [21] found that if parents participated in activities with their children often, communicated with their children often, and there was less family conflict, the onset of alcohol use by 11–18 year olds was delayed. These findings support a positive relationship between parental behavior and children's alcohol use and imply that certain parental behaviors may decrease the likelihood of children initiating alcohol use.

There is also evidence that the association between parents' drinking and children's alcohol use is mediated by family interaction variables. Moser & Jacob [25] found that dual and mother-only alcoholic families had impaired interaction. El-Sheikh, & Flanagan [29] confirmed that the association between parental problem drinking and children's externalizing problems was mediated by parent-child conflict. Latendresse et al [30] also demonstrated that parental monitoring and discipline are important mediators of the association between parental and adolescent alcohol use. Yu [31] found that parents' alcohol use did not have a direct effect on the time at which adolescents initiated alcohol use before the ages of 15–18. Rather, the shortened interaction time between children and parents was the reason behind the drop in age of onset of alcohol use. Parents' drinking may result in dysfunctional family relationships that constitute a noxious social environment for the children. Parents' alcohol use may have an effect on children's alcohol initiation through more family conflict or less parental support. Furthermore, White et al. [32] found that even after controlling for parents' proactive behavior, the effect of parents' alcohol use on children's alcohol use remained significant. This implies that both family interaction and parents' alcohol use are independently associated with children's first use of alcohol. Despite this volume of research, few studies to date have looked at the etiology of first alcohol use in elementary school children in a country with a low prevalence of drinking.

The overall aim of our study was to explore the effects of parents' alcohol use, parental support, and family conflict on the initiation of alcohol use in a sample of school children. We also examined whether the effect of parents' alcohol use and family interaction on children's initiation of alcohol were independent of each other.

## Methods

### Participants

Data for this study was obtained from the Child and Adolescent Behaviors in Long-term Evolution (CABLE) Project [33]. CABLE commenced in 2001 and is funded by the National Health Research Institutes in Taiwan. The CABLE student sample was established by randomly selecting nine schools each in Taipei City and Hsinchu County. Data were collected on the behaviors of school children in the first grade (cohort 1) and fourth grade (cohort 2) of these schools as well as their parents. After obtaining informed consent, children and their parents filled out separate self completed questionnaires. The two study cohorts were both followed up annually. The student questionnaires were completed by a whole class group at a time during a single lesson. For the parent questionnaires, students took the questionnaires home and asked their mothers and fathers to complete them separately. After completion, parents were asked to place the questionnaires in a sealed envelope, after which the children took them back to school and handed them in to a teacher.

In this study we analyzed data from the 2,499 students in cohort 2. In order to compensate for recall bias and to increase the accuracy of measurement of onset of alcohol use, we used school children's alcohol use data from 2002 (when students were 5^th ^graders and aged 10–11 years) and 2003 (when students were 6^th ^graders and aged 11–12 years.). Participants with missing data were excluded. There were a total of 1,571 children with complete questionnaire data for themselves and both parents for 2002 and 2003, which was 63% of the original sample. The chi-squared test was used to assess differences between the final and original samples in regards to demographic variables (sex, residential area, parents' marital status, father's education level, mother's education level and household monthly income). Those not included in the final sample were more likely to have parents that were not married or not living together (χ^2 ^= 45.9, p < 0.01), and were more likely to have a mother with a low education level (χ^2 ^= 7.7, p < 0.05). There were no differences in terms of sex, residential area (Taipei city, and Hsinchu county), father's education level, and household monthly income. After excluding the 388 students who were already using alcohol in 2002, final analyses were carried out on the remaining 1,183 children who were never users in 2002.

### Measures

Data on children's use of alcohol was taken from the 2002 and 2003 self completed student questionnaires. Data on parental support, family conflict, sex, and residential area were also taken from the 2002 self completed student questionnaires. The remaining data was obtained from the 2002 parent self completed questionnaires.

### Alcohol use by school children

Donovan [34] has suggested that in the measurement of initiation of alcohol use, first alcohol use should be considered as the first time alcohol is consumed by an individual who has previously never used alcohol (lifetime abstainer). We have used this suggested standard to define first alcohol use in our study. Use of alcohol was assessed by the question "Have you ever used alcohol?", and was scored using a Likert scale which in the 2002 questionnaire included the responses: 1. Never; 2. Yes, but not in the past month; 3. Once or twice in the past month; 4. Many times in the past month; or 5. Every day in the past month. In the 2003 questionnaire response options were: 1. Never; 2. Yes, but not in the past year; 3. Yes, but not in the past month; 4. Once or twice in the past month; 5. Many times in the past month; or 6. Every day in the past month. For both years, we divided the children into two groups based on their above responses: 1. Never-user; or 2. Ever-user. Changes in status between 2002 and 2003 were then used to divide the children into three alcohol use groups: Never-user (never-user in 2002 and 2003); First-time user (never-user in 2002 but ever-user in 2003); and Ever-user (ever-user in 2002 and 2003). Finally, we excluded those students who were ever-users initially before investigating influential factors on first alcohol use.

### Parent's alcohol use

Assessment of parent's alcohol use in the 2002 parent's questionnaire included the responses: 1. Never; 2. Yes, but not in the past month; 3. Once or twice in the past month; 4. Many times in the past month; or 5. Every day in the past month. We grouped responses 1 and 2 as never-users and responses 3, 4 and 5 as ever-users. Then based on the matching of alcohol use between both parents, a dummy variable including the following four groups was created: both parents ever-users; mother ever-user and father never-user; father ever-user and mother never-user; and both parents never-users. The reference group was both parents never-users.

### Family interaction factors

This included both parental support and family conflict. Parental support was measured by seven items, "Do your parents encourage you when you face difficulties", "Do your parents praise you for your achievements", "Do your parents console you when you are upset", "Do your parents care for you when you are sick", "Do your parents listen to what you say", "Do your parents care about what happens at school", "Do your parents help you solve problems". The internal consistency (Cronbach's alpha) for the study sample was 0.88. Family conflict was measured by the five items: "mum and dad argue", "mum and dad physically fight", "my brothers and sisters argue", "my brothers and sisters physically fight", "I argue with mum and dad or other family members". The Cronbach's alpha value for our sample was 0.60. All of the above items were scored from one (never) to four (every day). Higher mean scores on each measure indicated more parental support and more conflict. Factor analysis revealed the R^2 ^for these two variables was 58.57% in parental support and 39.30% in family conflict.

### Background factors

This included seven demographic dummy variables. Factors associated with alcohol use in past research [35-37] include sex, residential area, parents' marital status, and family socioeconomic status. Therefore relevant variables included in this study were: sex (female as the reference group), residential area (Taipei city and Hsinchu county; Taipei city as the reference group), parents' marital status (married and living together and other; other as the reference group), household monthly income (<40000, 40000–100000, >100000; >100000 as the reference group), father's education level (middle school and below, high school, university or research institute; university or research institute as the reference group), and mother's education level (middle school and below, high school, university or research institute; university or research institute as the reference group).

### Ethics

Ethics approval was given in 2001 by the Human Ethics Committee at National Health Research Institutes, Miaoli, Taiwan.

### Data analysis

Data analysis was carried out using SPSS 12.0 statistical software. Descriptive statistical analysis included mean values, percentages and frequency distributions. The dependent variable was alcohol use group and the independent variables were background factors, parent's alcohol use, parental support and family conflict. Bivariate analysis was used to examine the correlations between each variable and alcohol use group. Stepwise logistic regression was then used to assess the relationship between independent variables and first alcohol use in school children.

Two separate regression models were built. In the first step of the analysis, we entered background variables and parents' alcohol use factors into model 1 to test the effect of parents' alcohol use on children's first alcohol use. In the second step, we constructed the full model that included all independent variables to explore the effect of parental support and family conflict on the children's first alcohol use. This allowed us to examine the changes in significance of parents' alcohol use after the addition of these two family interaction factors.

## Results

### Alcohol use and predictors in the study sample

There were 1,000 students (84.5%) who were never-users of alcohol in both grade 5 and grade 6 and 183 students (15.5%) who were never-users in grade 5 but became first-time users in grade 6.

The distribution of predictors in the study sample in each of the alcohol use subgroups are shown in Table 1. In general, 46.1% were boys and 53.9% were girls; and 54% lived in a metropolitan area (Taipei city). In regards to the parents, 93.7% of parents were married and living together; 55.5% had monthly household incomes of NT$40,000 to NT$100,000. The majority of parents had a high school level of education (57% of fathers and 66.3% of mothers). Parents who were both never-users of alcohol constituted 44.4% of the sample. First-time alcohol users were more likely to have a lower mean score for parental support (22.1 ± 4.9 vs 20.8 ± 5.3, t = 3.36, p < 0.01), and to have a higher mean score for family conflict (8.8 ± 1.9 vs 9.2 ± 2.1, t = -2.63, p < 0.01).

**Table 1:** Predictors of alcohol use in the study sample

	Total		Alcohol use group					
			
Variable	(n = 1183) n % mean ± SD		Never-user (n = 1000) n % mean ± SD		First-time user (n = 183) n % mean ± SD			
Sex							χ^2 ^= 2.45	
Male	545	46.1	451	45.1	94	51.4		
Female	638	53.9	549	54.9	89	48.6		
Residential area							χ^2 ^= 0.12	
Taipei	639	54.0	538	53.8	101	55.2		
Hsinchu	544	46.0	462	46.2	82	44.8		
Parents' marital status/living arrangements							χ^2 ^= 0.34	
Married and live together	1109	93.7	938	93.8	171	93.4		
Other	74	6.3	62	6.2	12	6.6		
Household monthly income (NTD)								
<40000	173	14.6	156	15.6	17	9.3	χ^2 ^= 5.23	
40000–100000	657	55.5	552	55.2	105	57.4		
>100000	353	29.8	292	29.2	61	33.3		
Father's education level							χ^2 ^= 5.80	
Middle school and below	156	13.2	142	14.2	14	7.7		
High school	674	57.0	563	56.3	111	60.7		
University or research institute	353	29.8	295	29.5	58	31.7		
Mother's education level							χ^2 ^= 1.72	
Middle school and below	165	13.9	138	13.8	27	14.8		
High school	784	66.3	670	67.0	114	62.3		
University or research institute	234	19.8	192	19.2	42	23.0		
Parents' matched alcohol use group							χ^2 ^= 5.56	
Both parents ever-users	167	14.1	135	13.5	32	17.5		
Mother ever-user, father never-user	60	5.1	48	4.8	12	6.6		
Father ever-user, mother never-user	431	36.4	360	36.0	71	38.8		
Both parents never-users	525	44.4	457	45.7	68	37.2		
Parental support (score 7–28)	21.9 ± 5.0		22.1 ± 4.9		20.8 ± 5.3		t = 3.36	**
Family conflict (score 5–20)	8.8 ± 2.0		8.8 ± 1.9		9.2 ± 2.1		t = -2.63	**

**p < .01

Never-user defined as never used alcohol in 2002 and 2003, first-time user defined as never used alcohol in 2002 but ever used alcohol in 2003

Table 2 presents the correlation matrix for the variables. Of note is that alcohol use group was significantly positively correlated with household income, parents' alcohol use, and family conflict but significantly negatively correlated with parental support. In addition, family conflict was significantly negatively correlated with parental support (r = -0.20). However, parents' alcohol use was not significantly associated with family interaction factors, either parental support or family conflict.

**Table 2:** Correlation matrices for variables

variable	1	2	3	4	5	6	7	8	9	10
1. Sex^a^	1									
2. Area^b^	-0.03	1								
3. Parent's marital status^c^	0.00	-0.06*	1							
4. Household income^d^	0.06*	-0.30***	0.08**	1						
5. Father's education level^e^	0.04	-0.31***	0.09**	0.46***	1					
6. Mother's education level^f^	0.02	-0.30***	0.07*	0.46***	0.57***	1				
7. Parents' matched alcohol use group^g^	0.04	0.08**	-0.01	-0.03	-0.06*	-0.04	1			
8. Parental support	-0.13***	-0.08**	0.03	0.08**	0.12***	0.10***	-0.05	1		
9. Family conflict	-0.03	0.12***	-0.00	-0.08**	-0.04	-0.05	0.02	-0.20***	1	
10. Alcohol use group^h^	0.05	-0.01	-0.01	0.06*	0.05	0.02	0.06*	-0.10**.	0.08**	1

*p < .05; **p < .01; ***p < .001

^a^Sex 0 = Female; 1 = Male

^b^Area 0 = Taipei; 1 = Hsinchu

^c^Parent's marital status 0 = other; 1 = married and live together

^d^Household income(NTD) 1 =< 40000; 2 = 40000–100000; 3 => 100000

^e^Father's education level 1 = Middle school and below; 2 = High school; 3 = University or research institute

^f^Mother's education level 1 = Middle school and below; 2 = High school; 3 = University or research institute

^g^Parents' matched alcohol use group 1 = Both parents never-users; 2 = Mother ever-user, father never-user; 3 = Father ever-user, mother never-user; 4 = Both parents ever-users

^h^Alcohol use group 0 = Never-user; 1 = First-time user

### Factors related to first-time alcohol use in sixth grade children

Logistic regression was used to determine factors associated with first-time alcohol use in sixth graders. The results are shown in Table 3. Model 1 revealed that without considering parental support and family conflict, parents' alcohol use increased the odds of children's initiation of alcohol use. Alcohol use by both parents was significantly associated with first time alcohol use by sixth grade students (OR:1.62, 95%CI = 1.01–2.59). A non-significant relationship was found between having only one parent who was an ever-user (father or mother) and first use of alcohol in the sixth grade. Model 2 showed that after adjusting for parents' alcohol use, children with less perceived parental support (OR: 0.95, 95%CI = 0.92–0.99) and more family conflict (OR: 1.11, 95%CI = 1.02–1.20) were more likely to become first-time users of alcohol. Moreover, after adjusting for family interaction factors, the effect of parents' alcohol use on children's first use of alcohol remained significant (OR:1.64, 95%CI = 1.02–2.64).

**Table 3:** Factors associated with first alcohol use in the study sample (n = 1183)

	Model 1			Model 2		
		
Variable	β	OR	95%CI	β	OR	95%CI
Sex: male/female	0.19	1.20	0.88–1.66	0.13	1.14	0.82–1.59
Area: Hsinchu/Taipei	0.02	1.03	0.72–1.46	-0.05	0.96	0.67–1.37
Parent's marital status/living arrangements: Married and living together/other	-0.17	0.84	0.44–1.62	-0.18	0.83	0.43–1.61
Household income (NT$/month):						
<40,000/>100,000	-0.59	0.55	0.29–1.07	-0.68*	0.51	0.26–0.98
40,000–100,000/>100,000	-0.10	0.91	0.61–1.36	-0.12	0.89	0.59–1.33
Father's education level						
Middle school and below/university or research institute	-0.62	0.54	0.26–1.12	-0.70	0.50	0.24–1.05
High school/university or research institute	0.11	1.11	0.71–1.75	0.12	1.12	0.71–1.78
Mother's education level						
Middle school and below/university or research institute	0.27	1.32	0.67–2.59	0.27	1.31	0.66–2.61
High school/university or research institute	-0.23	0.80	0.49–1.30	-0.27	0.77	0.47–1.25
Parents' matched alcohol use group						
Both parents ever-users/both never-users	0.48*	1.62	1.01–2.59	0.50*	1.64	1.02–2.64
Mother ever-user, father never-user/both never-users	0.50	1.65	0.83–3.27	0.46	1.58	0.79–3.18
Father ever-user, mother never-user/both never-users	0.25	1.28	0.89–1.85	0.21	1.24	0.86–1.79
Parental support (score 7–28)				-0.05**	0.95	0.92–0.99
Family conflict (score 5–20)				0.10*	1.11	1.02–1.20

*p < .05; **p < .01

These findings suggest that parents' alcohol use, family conflict and parental support all play important roles in the risk of initiation of alcohol use by children.

## Discussion

The earlier that young people start drinking, the more likely they are to misuse alcohol and to experience alcohol dependence and alcohol-related unintentional injuries later in life [2,38,39]. Our results highlight the role of parents' alcohol use and family interaction in the progression from abstention to first alcohol use in sixth grade children. Past researchers [7,12,19,22,26] have examined the factors related to the initiation of alcohol use. In our study we have followed the definition of Donovan [34], which more strictly defines first alcohol use and is more suitable for school children in countries with a low rate of alcohol use. After excluding those children who had already used alcohol, our study found that 15.5% of children became first-time users in the sixth grade. As the Children and Youth Welfare Law [40] prohibits any consumption of alcohol by children, any increase in the proportion of children with first alcohol use in the sixth grade warrants attention.

The family is a child's first social environment and provides the child with a background for behavioral development of both risky and healthy behaviors. Although other research [32,41] has found that father's alcohol use and mother's alcohol use have different influential effects on adolescents' drinking behavior, our results did not find any individual effects of father's or mother's alcohol use. The likelihood of children initiating alcohol use was only increased when both parents were using alcohol. In contrast to other studies, our data was obtained from parents' self reports, and therefore is more able to clarify the influence of parents' alcohol use on school children's initiation of alcohol use. Our findings agree with those of Li et al. [23], who found that children's alcohol use was influenced by the number of parents using alcohol. According to the social development model [16], the behavior of an individual will be prosocial or antisocial depending on the predominant behaviors, norms, and values held by those individuals or institutions to which the individual is bonded. It is possible that having both parents use alcohol provides children with more opportunities for observation and reinforcement of this behavior [17], such as the imitation of their parents' drinking behavior, internalization of their parents' attitudes of support for alcohol use, and positive expectations of the effects of alcohol [16,28,42].

Cultural differences might be another explanation for the differences that we have observed. American and European culture is more individualistic as opposed to Chinese culture, which is more collectivistic than European, African, American, and other Asian cultures [43]. Collectivist societies emphasize the social unit with a common fate. Common values are centralized rather than promoting personal uniqueness and focusing on the individual as in individualistic societies. Family is the primary society for the individual, and having a father and mother who both drink may send a stronger impression of the social context of acceptable drinking behavior to children than the effect of only one parent drinking. Warner and White [8] found that many children and adolescents first use alcohol at family gatherings. Having a father and mother who both use alcohol may increase children's contact with alcoholic drinks and their opportunities to taste alcohol at family gatherings.

After controlling for parental support and family conflict, parent's alcohol use still had a significant effect on sixth graders' first alcohol use. This result agrees with the findings of previous research [19-21]. We did not find a relationship between parents' drinking and family interaction, either parental support or family conflict. Parents' drinking in this study was not defined according to quantity or the presence of a drinking problem as in previous studies [25]. It is possible that the association of parents' drinking with parental support and family conflict occur only with heavier alcohol use by parents. Our results are similar to the position of White et al [32] who found that the family factors of father's and mother's alcohol use, and parental warmth or hostility had independent effects on children sustaining alcohol use from adolescence into adulthood. It is implied that both family interaction and parents' drinking are equally important in preteen's first use of alcohol.

Our results agree with those of other researchers [21,22,26] who have found that parental emotional support is an important protective factor against the initiation of alcohol use by adolescents and that children whose parents communicate with them more often are less likely to begin using alcohol in adolescence. The measurement instrument for family support in our study incorporated parental listening and care which may reflect the amount of time parents spend communicating with their children. Research [44,45] indicates that frequent communication and involvement between parents and children can increase adolescents' ability to say no to alcohol in a variety of situations and as a result decreases the influence of friends with unhealthy behavior and decreases substance use behavior. Our finding that family conflict can predict alcohol initiation in sixth grade children also agrees with previous research [27,28]. Social bonds to conventional society inhibit association with delinquent peers and, in turn, prevent delinquent behavior [16]. Children who experience more family conflict are more likely to use alcohol because their parents show less concern about their whereabouts or because they lack social skills for coping with stress. This enables these children to both congregate with other peers that have unhealthy behaviors and carry out unhealthy behavior themselves [27,28].

For the most part, our findings replicate those from the United States. This could be due to the high level of westernization of the Taiwanese study population, or alternatively it could be due to common social psychological processes that transcend cultural differences in causing or buffering against alcohol use among preteens.

Decreasing the proportion of children and adolescents using alcohol and delaying the age of onset of alcohol use are important goals for alcohol use policy [1,10]. Our study makes three major contributions to this field of research. Firstly, our study subjects were a number of elementary school students whose alcohol use behavior was followed over two years. This enabled the confirmation of a group of children who changed from being an abstainer to a first-time user, and allowed us to examine risk factors for sixth graders' first use of alcohol. Secondly, having parents who were both alcohol users significantly increased the likelihood of school children initiating alcohol use. It is possible that having a proactive family relationship could decrease the likelihood of initiation of alcohol use by school children. Based on our findings, we recommend the strengthening of prevention efforts targeting parents, focusing on educating them about the effects of alcohol use on children and about proactive family management practice. Finally, our study furthers understanding of underage alcohol use in a low alcohol consumption society, and similar research might be replicated in other low alcohol use countries.

Limitations of our study include changes to how alcohol use was measured over the two years. This meant that we were only able to explain factors predicting school children's first alcohol use in the sixth grade. Future research should conduct longer follow up in order to increase the understanding of factors influencing alcohol use behavior in children. Secondly, due to data limitations we were unable to investigate the amount of alcohol consumed by parents or children or the type of alcohol consumed. However, as the legal age for drinking in Taiwan is 18 years, and our study was mainly concerned with factors influencing initiation of alcohol use amongst school children, we feel that this is not a major concern. Finally, even though family conflict was significantly associated with preteen's first alcohol use, it is important to note that the Cronbach's alpha coefficient for family conflict was quite low. 

## Conclusion

In conclusion, parent's alcohol use, parental support and family conflict had an influential effect on the initiation of alcohol use by sixth grade children. We recommend the strengthening of prevention efforts targeting parents' family management practices and emphasizing the unfavorable effect of parents' alcohol use on preteen early use of alcohol.

## Competing interests

The authors declare that they have no competing interests.

## Authors' contributions

CCH designed the study, carried out statistical data analysis, interpreted the data, and prepared the manuscript; LLY contributed to design development, data interpretation, and provided data analysis advice; WCW helped to perform the statistical analysis as well as to prepare the draft of the manuscript. All authors read and approved the final manuscript.

## Pre-publication history

The pre-publication history for this paper can be accessed here:


